# Hypophosphatasia: A Novel Mutation Associated with an Atypical Newborn Presentation

**DOI:** 10.4274/jcrpe.galenos.2019.2018.0263

**Published:** 2020-03-19

**Authors:** Roger Esmel-Vilomara, Susana Hernández, Ariadna Campos-Martorel, Eva González-Roca, Diego Yeste, Félix Castillo

**Affiliations:** 1 Hospital Universitari Vall d’Hebron, Clinic of Pediatrics, Barcelona, Spain; 2 Hospital Universitari Vall d’Hebron, Clinic of Neonatology, Barcelona, Spain; 3 Hospital Universitari Vall d’Hebron, Clinic of Pediatric Endocrinology, Barcelona, Spain; 4 Hospital Clínic de Barcelona, Clinic of Immunology, Barcelona, Spain

**Keywords:** Hypophosphatasia, mutation, newborn, alkaline phosphatase

## Abstract

Hypophosphatasia, a rare genetic disease affecting bone metabolism, is characterized by decreased activity of tissue non-specific alkaline phosphatase (TNAP). The gene encoding TNAP *(ALPL)* has considerable allelic heterogeneity, which could explain different degrees of enzyme activity resulting in a wide clinical variability. We report the case of a preterm newborn in whom a corneal opacity was detected at birth. Blood tests performed to investigate this finding showed low alkaline phosphatase concentrations. The corneal opacity disappeared within a week but alkaline phosphatase remained persistently low. With persistently decreased levels of alkaline phosphatase, upon suspicion of hypophosphatasia, plain radiography detected changes suggestive of rickets. Sequencing of the *ALPL* gene revealed a heterozygous variant that has not been described in the literature to date. Our patient’s condition may be an atypical neonatal form of the syndrome, with a mild phenotype, very different from the classic neonatal form, which can lead to severe skeletal disease and respiratory failure. However, it could also be an early diagnosis of the childhood form, which is associated with a better prognosis.

**What is already known on this topic?**

Hypophosphatasia is a rare Mendelian disease affecting bone metabolism through loss-of-function mutations in the gene encoding the tissue non-specific isoenzyme of alkaline phosphatase. To date, 381 different mutations have been described. The clinical expression of the disorder varies widely and can even differ between patients with the same mutation.

**What this study adds?**

This report describes a novel mutation that has not been described in the literature to date. Also described is an atypical neonatal presentation of the syndrome, with mild phenotype, which is different to the classic neonatal form. However, it could also be an early diagnosis of the childhood form, which has better prognosis.

## Introduction

Hypophosphatasia is a rare Mendelian disease affecting bone metabolism, in which activity of the tissue non-specific isoenzyme of alkaline phosphatase (TNAP) is decreased because of loss-of-function mutations in the gene encoding this isoenzyme (1,2). In Europe the incidence of severe forms has been estimated at 1/300,000 newborns. The prevalence of mild forms has not been reported, but is expected to be higher (3).

The clinical expression of hypophosphatasia varies widely, from death *in utero *with an unmineralized skeleton to problems with dentition in adult life, or an absence of symptoms (4). The severity of the disorder can even differ between patients with the same mutation (1). There are six forms of hypophosphatasia. Four are classified according to the age when bone disease becomes apparent: perinatal; infantile; childhood; and adult; with early-onset forms being the most severe. Odontohypophosphatasia exclusively causes dental manifestations. Some authors also include pseudohypophosphatasia, a rare infantile form with normal alkaline phosphatase values (4,5). The last type is benign prenatal hypophosphatasia, similar to the perinatal form in the age at presentation, but with a better prognosis and spontaneous clinical improvement (1).

We describe the case of an infant with an unusual presentation of hypophosphatasia diagnosed shortly after birth during investigation of an incidental clinical finding. A previously unreported mutation, found in the gene (*ALPL*) encoding TNAP, may be the cause of the patient’s mild form of the disorder.

## Case Report

A late preterm male infant (34+3 weeks) was admitted to the minimal care neonatal unit for prematurity and infection risk, due to delivery five days after premature rupture of membranes. Cleft palate had been detected in the second trimester ultrasound study. The patient’s family background included three paternal family members with cleft palate.

A corneal opacity was detected at birth. On blood testing to investigate this finding at five days after birth, the phosphate concentration was 8.2 mg/dL (normal value in preterm infants <7.9 mg/dL) with normal calcium (9.6 mg/dL), vitamin D (25.4 ng/mL) and parathyroid hormone (19 pg/mL) but alkaline phosphatase was low at 52 IU/L (normal value >75 IU/L). By one week of life, the corneal opacity had disappeared and the infant was asymptomatic.

In subsequent analyses, alkaline phosphatase remained low up to a maximum of 67 IU/L, with phosphate value below 8 mg/dL. The main potential causes of low alkaline phosphatase - hypothyroidism, anemia, low magnesium or zinc concentrations, vitamin D intoxication, low vitamin C, Wilson’s disease, or intake of certain drugs - were ruled out. This led to a possible diagnosis of hypophosphatasia.

**Figure 1:**
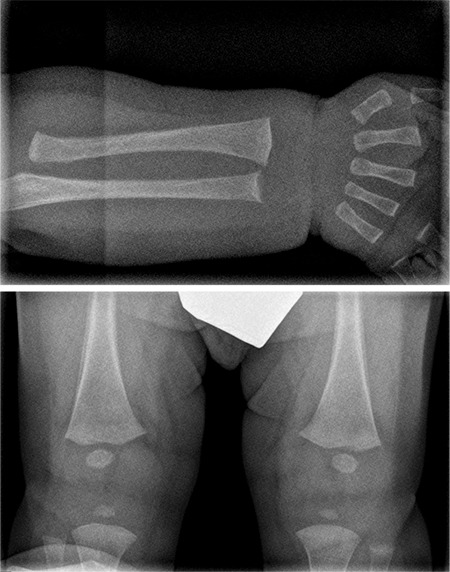
Radiographs at the time of the hypophosphatasia diagnosis

Radiographic studies on day eight of life revealed low radiological bone density. Subsequent radiography at 2.5 months of life (Figure 1) showed irregularities and widening of the radial and ulnar metaphyses, suggestive of rickets. A periosteal reaction in the femur with discrete metaphyseal widening was also observed.

Molecular genetic studies were performed using polymerase chain reaction amplification with specific primers. The sequencing reaction was carried out with the BigDye Terminator v3.1 Sequencing Kit (Applied Biosystems, Foster City, California, USA), capillary electrophoresis was performed on an ABI Prism 3730xl DNA Analyzer Sequencer (Applied Biosystems), and chromatograms were generated with the SeqPilot software (JSI Medical Systems, Ettenheim, Germany).

A heterozygous variant, c.1292T>A, was found in exon 11 of the *ALPL* gene, producing an amino acid change: p.(Val431Asp). This specific mutation, in the gene affected in hypophosphatasia, had not previously been reported (6). Bone densitometry of the lumbar spine was performed which showed a bone mineral density of 0.359 g/cm^2^, consistent with normal reference values for Spain.

Clinical evaluation of first-degree relatives to investigate possible affected individuals within the family was negative, but the patient’s mother showed a low plasma concentration of alkaline phosphatase (28 IU/L). She did not present stigmata of rickets or early tooth loss and her height was within the limits of normality. The gene encoding TNAP was analyzed to determine whether the mutation was *de novo* or inherited, and the same mutation was found in the mother.

**Figure 2:**
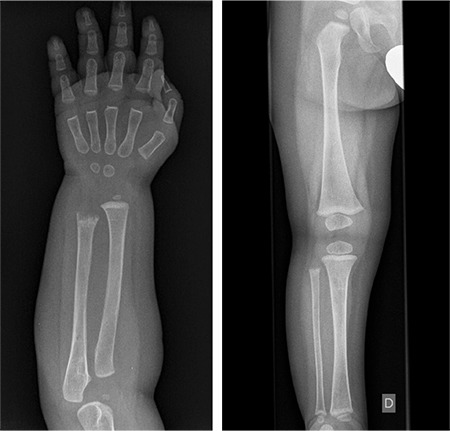
Radiographs at the age of 14 months

Currently, the patient is being monitored with periodic follow-up visits and is not receiving replacement therapy. Two years after diagnosis the alkaline phosphatase values remain low and are always <70 IU/L. Radiographic studies (Figure 2) show signs of diffuse osteopenia with affected distal metaphysis of both ulnas with marked deflection, loss of trabecular density and slight significant widening on the left ulna. The rest of the metaphysis are enlarged with only a slight sclerotic line on the physis. There are no other skeletal findings.

Written informed consent was obtained from the patient’s parents for publication of this case report and the accompanying images.

## Discussion

Hypophosphatasia is a rare inherited disease characterized by decreased alkaline phosphatase levels due to reductions in the tissue non-specific isoenzyme. Presumptive diagnosis of this disease is based on physical examination and consistent radiographic findings, in association with low concentrations of alkaline phosphatase in blood tests. The molecular diagnosis is established by sequencing the gene encoding TNAP (1).

The casual finding of corneal opacities in this patient was the starting point of the diagnosis, although the opacities had disappeared by the end of the first week of life. An association between hypophosphatasia and ocular signs of hypercalcemia, morphologically identical to band keratopathy, has been reported (7). However, the fast, spontaneous resolution of this finding suggests that it was not an abnormality related with the genetic disease.

This case exemplifies an atypical presentation of the disorder, as the patient was asymptomatic at the time of the diagnosis, but had low alkaline phosphatase levels. Physical examination at birth provided no evidence of hypophosphatasia and radiographs only detected non-specific osteopenia. However, in subsequent examinations, the patient had the characteristic decrease in alkaline phosphatase activity and radiographic findings suggestive of hypophosphatasia. Imaging typically shows a generalized decrease in bone density and metaphyseal abnormalities in long bones, similar to those found in severe forms of rickets (1,2). However, unlike rickets, serum alkaline phosphatase levels are decreased in hypophosphatasia (1,4).

Reported evidence has indicated that the lower the alkaline phosphatase levels, the more severe are the manifestations of the disorder (1,8). The mutations in severe hypophosphatasia produce a protein that fails to reach the cell membrane, but instead, accumulates in the cis-Golgi apparatus and is then degraded in the proteasome without producing adequate enzymatic activity. However, mutations found in the mild forms produce enzymes that are, in part, properly located at the cell membrane and exhibit significant residual activity (9).

Our patient did not have very low levels of the enzyme, which suggests that the newly identified genetic variant may cause a mild phenotype. It may be an atypical, mild neonatal form, considering the age of presentation and absence of clinical features, despite the presence of radiographic abnormalities. Nonetheless, we cannot exclude that it may represent an early diagnosis of childhood hypophosphatasia, with a better prognosis than the neonatal forms. It cannot be considered as benign prenatal hypophosphatasia, as the diagnosis was made after birth and the patient had no visible abnormalities on prenatal ultrasound studies apart from the cleft palate.

The definitive diagnosis of hypophosphatasia is established by sequencing the *TNAP* gene, *ALPL*, located on chromosome 1p36.1-p34 (1,6), and subject to very strong allelic heterogeneity. To date, 381 different mutations have been described in *ALPL*, 70.6% of which are missense mutations (6). Allelic heterogeneity explains the different degrees of enzyme activity and the great variability observed in the clinical expression of the disease (1,3). In our case, a previously unidentified mutation, a heterozygous variant in exon 11 of *ALPL* (c.1292T>A) was detected in both the patient and his mother. This mutation produces an amino acid change: p.(Val431Asp). Bioinformatic studies using SIFT (10) and Polyphen2 algorithms (11) predicted a harmful effect of the mutation on the structure or function of the protein. According to the American College of Medical Genetics and Genomics guidelines for the interpretation of sequence variants (12), the mutation would be considered probably pathogenic.

As to inheritance, severe forms of hypophosphatasia (perinatal and infantile) have an autosomal recessive transmission, whereas in mild forms, transmission can be recessive or dominant (2). Sporadic cases are rare. Our patient had a heterozygous mutation, suggesting dominance. The negative effects of some heterozygous mutations, resulting in TNAP activity from 20 to 40% of wild-type, explain the dominant transmission of hypophosphatasia. A mild phenotype is expected in the heterozygous state even if the mutation has a severe effect (1,2).

The dominant negative effect is corroborated by a previous missense mutation in the position of Val431. A mutation in the same allele -p.(Val431Ala)- leading to a change from valine to alanine, has been described in relation with odontohypophosphatasia (6). The clinical form of the condition in our patient remains to be defined, but as bone was affected on radiography, it is unlikely to be odontohypophosphatasia.

The severity of the condition can differ between patients showing the same mutation (1), and there is significant variability in the expected clinical features (3). In our patient, the mutation has a putative dominant inheritance; hence, relatives with the mutation might or might not develop mild hypophosphatasia and the patient’s mother is still asymptomatic. It could be that this patient and his mother will have low alkaline phosphatase levels and harbor a recessive variant in the TNAP allele but never experience symptoms, in which case they could be considered carriers (1). The variability in the inheritance models and the variable penetrance complicates genetic counseling, although it could be of value in couples with an affected child (2,3).

Considering that the accumulation of the enzymatic substrates will be a key point in the future follow-up (1,4), to be able to predict any clinical manifestations of progression of the disease, the next step to confirm the phenotype would be the determination of the substrates of the enzyme.

Asfotase alfa, a recombinant human tissue non-specific alkaline phosphatase coupled to a deca-aspartate motif for bone targeting, has demonstrated efficacy for healing the skeletal manifestations of hypophosphatasia. In addition, it has been found to improve respiratory and motor function in perinatal and infantile forms, with a good safety profile (13). Given the age of the patient, the disease course should be monitored so that management can be adapted to the abnormalities that may occur.

A previously unidentified heterozygous variant located in exon 11 of the *ALPL* gene -c.1292T>A- is described as a cause of hypophosphatasia.

The case reported could be considered an atypical presentation of the neonatal form, with a mild phenotype and different from the classical neonatal form, or the childhood form with a very early diagnosis, which is associated with a better prognosis. The child’s condition should be closely monitored, and if deemed appropriate, replacement therapy with asfotase alfa can be provided.

Persistently low levels of alkaline phosphatase should alert clinicians to hypophosphatasia. Definitive diagnosis can be achieved by genetic analyses.
